# Amygdala Adaptation and Temporal Dynamics of the Salience Network in Conditioned Fear: A Single-Trial fMRI Study

**DOI:** 10.1523/ENEURO.0445-17.2018

**Published:** 2018-02-28

**Authors:** Siyang Yin, Yuelu Liu, Nathan M. Petro, Andreas Keil, Mingzhou Ding

**Affiliations:** 1 J. Crayton Pruitt Family Department of Biomedical Engineering, University of Florida, Gainesville, FL 32611; 2 Center for Mind and Brain, University of California, Davis, CA 95618; 3 Department of Psychology, University of Florida, Gainesville, FL 32611

**Keywords:** adaptation, amygdala, fear conditioning, salience network

## Abstract

Research in rodents has established the role of the amygdaloid complex in defensive responses to conditioned threat. In human imaging studies, however, activation of the amygdala by conditioned threat cues is often not observed. One hypothesis states that this finding reflects adaptation of amygdaloid responses over time. We tested this hypothesis by estimating single-trial neural responses over a large number of conditioning trials. Functional MRI (fMRI) was recorded from 18 participants during classical differential fear conditioning: Participants viewed oriented grayscale grating stimuli (45° or 135°) presented centrally in random order. In the acquisition block, one grating (the CS+) was paired with a noxious noise, the unconditioned stimulus (US), on 25% of trials. The other grating, denoted CS–, was never paired with the US. Consistent with previous reports, BOLD in dorsal anterior cingulate cortex (dACC) and insula, but not the amygdala, was heightened when viewing CS+ stimuli that were not paired with US compared to CS– stimuli. Trial-by-trial analysis showed that over the course of acquisition, activity in the amygdala attenuated. Interestingly, activity in the dACC and insula also declined. Representational similarity analysis (RSA) corroborated these results, indicating that the voxel patterns evoked by CS+ and CS– in these brain regions became less distinguishable over time. Together, the present findings support the hypothesis that the lack of BOLD differences in the amygdaloid complex in many studies of classical conditioning is due to adaptation, and the adaptation effects may reflect changes in large-scale networks mediating aversive conditioning, particularly the salience network.

## Significance Statement

In neuroimaging studies of human fear conditioning, activation of the amygdala is often not observed. This problem is examined by applying single-trial functional MRI (fMRI) analysis to a classical fear conditioning paradigm. In addition to confirming the amygdala adaptation hypothesis, we discovered that areas of the salience network, to which the amygdala belongs, coadapted, supporting the hypothesis that the acquisition of defensive responses in humans is mediated by changes in a large-scale brain network.

## Introduction

In classical fear conditioning, an initially neutral stimulus (conditioned stimulus, CS+), through repeated associations with an aversive stimulus, acquires the ability to elicit defensive responses in the absence of the original aversive stimulus. Research in rodents has implicated the amygdala as a key neural substrate that mediates the acquisition of fear (LaBar et al., 1995; LaBar and LeDoux, 1996), especially during the early stages of fear acquisition (Ono et al., 1995; Blair, 2008). Human imaging studies, however, have not always observed activation of the amygdala in fear conditioning paradigms (Knight et al., 1999, 2004a; Phelps et al., 2004). One explanation is that BOLD responses in the amygdala adapt over time (Büchel et al., 1998). While some studies support this notion (LaBar et al., 1998; Morris et al., 2001), others did not find such adaptation effects (Critchley et al., 2002; Knight et al., 2004b; Merz et al., 2014). In the present study, our first goal was to examine the temporal dynamics of the amygdala by applying single-trial functional MRI (fMRI) analysis to an aversive conditioning paradigm, in which a large number of trials were included to specifically test the adaptation hypothesis.

Whereas amygdala activation during processing of CS+ has been less reliably observed, the activation of other brain regions, such as dorsal anterior cingulate cortex (dACC) and insula, is consistently reported (Sehlmeyer et al., 2009; Fullana et al., 2016). Recent work proposes that dACC and insula, together with a set of limbic and subcortical structures (including the amygdala), constitute the brain’s salience network (Seeley et al., 2007). This network is thought to mediate the detection and integration of behaviorally relevant stimuli (Menon and Uddin, 2010) including stimuli that elicit fear (Liberzon et al., 2003; Zheng et al., 2017). The question is, to what extent the other nodes of the salience network show similar adaptation dynamics as the amygdala, and if these dynamics reflect habituation of defensive engagement (Ishai et al., 2004). One previous study (Straube et al., 2007) compared early and late trials in acquisition and found decreasing CS+ related responses in the amygdala, but not in other CS+ activated regions. In the present study, our second goal was to investigate amygdala adaptation in the context of network dynamics, by extending single-trial BOLD analyses to other structures of the salience network, and by comparing adaptation dynamics across different indices extracted from the single-trial BOLD time course.

The advent of the multivariate pattern analysis (MVPA) in fear conditioning studies has complemented univariate approaches by demonstrating differences in spatial activation patterns evoked by CS+ and CS– (Visser et al., 2011; Dunsmoor et al., 2014). These studies, typically employing complex objects (faces, scenes, etc.) as CS+ and CS– and small numbers of trials, have shown that contingency acquisition results in increasing pattern similarity between exemplars within a CS type (i.e., CS+, CS–), even if these exemplars belong to different categories such as houses or faces (Visser et al., 2011, 2013, 2016). Recognizing that small numbers of trials may not allow the conditioning dynamics to fully unfold and that complex stimuli make the identification of pattern changes specifically related to fear learning challenging, in the present study, our third goal was to apply representational similarity analysis (RSA) to experimental designs that take these issues into consideration.

fMRI was recorded from participants performing a classical aversive conditioning experiment in which grayscale grating stimuli (45° or 135°) were used as CS+ and CS–. Single-trial level BOLD activities were estimated by the β-series method. The resulting time courses over a large number of acquisition trials were characterized and compared. In addition, RSA was applied to quantify pattern similarity between CS+ and CS–, and yield pattern similarity dynamics that were then compared to the univariate BOLD effects. We hypothesized that amygdala activity adapted over the course of acquisition, and that amygdala adaptation was accompanied by a decrease in the discriminative voxel patterns seen in structures known to possess strong connectivity with the amygdaloid, such as dACC and insula.

## Materials and Methods

### Experimental procedure

#### Participants

The experimental protocol was approved by the Institutional Review Board (IRB) of the University of Florida. Eighteen healthy college students (aged 17–33 years, nine females) provided written informed consent and participated in the study. The participants were either paid or given course credits in accordance with IRB guidelines.

#### Stimuli

Two Gabor patches (sine wave gratings filtered with a Gaussian envelope, Michelson contrast = 1) with the same spatial frequency (∼1.5 cycles/degree), differing only in orientation (45° and 135°), were designated as CS+ and CS–. Both stimuli were projected onto a back-illuminated screen (60 × 60 cm) placed ∼230 cm away from the subject’s head and viewed through a set of prismatic glasses attached to the radio frequency birdcage coil. The unconditioned stimulus (US) was a 1-s human scream delivered by a MRI compatible headphone at around 95 dB. For CS– trials and CS+ trials where CS+ and US were not paired, the Gabor patches were shown for 1 s. For CS+ trials where CS+ and US were paired, the US started 0.5 s following CS+ onset and both coterminated 1 s later.

#### Paradigm

**Figure 1.**
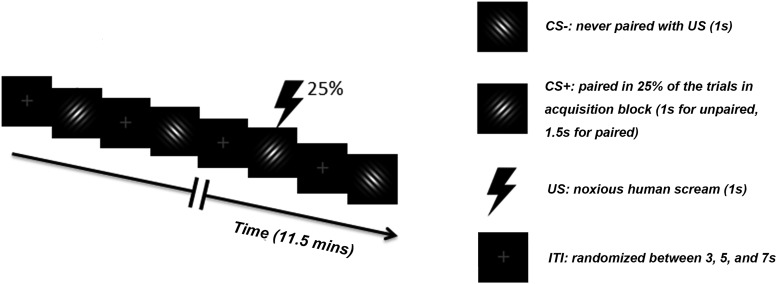
Timeline and stimuli used during the acquisition block. For the habituation and extinction blocks, stimulus types, stimulus duration, and ITIs were the same, except that no US was presented.

The experiment consisted of three blocks: habituation, acquisition and extinction; timeline of the acquisition block was depicted in Figure 1. Each block comprised 120 trials and lasted ∼12 min. In the initial habituation block, each Gabor patch was presented for 1 s, and the two Gabor patches occurred with equal probability in a pseudo-random order. Note that a substantial number of previous studies have not used habituation blocks, which makes the estimation of a priori differences difficult, but may also facilitate more rapid conditioning, compared to designs with initial presentations of both CSs without US exposure. During the acquisition block, one Gabor patch was designated as CS+ and the other as CS–. The first 4 CS+ trials were always paired with US to facilitate contingency acquisition. Following that, 25% of CS+ trials were paired with US, and the remaining 75% of CS+ trials were not. CS– trials were never paired with US. For notational simplicity, in what follows, we refer to CS+ trials where CS+ was not paired with US simply as CS+ trials. CS+ paired trials were not included in the analyses of CS+ activation due to contamination of CS+ responses by US evoked activities. In the extinction block, the stimuli and procedure were the same as the habituation block. For each of the three blocks the intertrial interval (ITI) was randomized with a mean ITI of 4.6 ± 1.5 s. The data recorded during the extinction block was not analyzed in this study.

### Data acquisition

#### fMRI data

fMRI images were acquired on a 3-Tesla Philips Achieva whole body MRI system (Philips Medical Systems) using a T2*-weighted echoplanar imaging (EPI) sequence [echo time (TE) = 30 ms; repetition time (TR) =1980 ms; flip angle = 80°]. Each whole-brain volume consisted of 36 axial slices (field of view: 224 mm; matrix size: 64 × 64; slice thickness: 3.50 mm; voxel size: 3.5 × 3.5 × 3.5 mm). A T1-weighted high resolution structural image was also obtained from each subject.

#### Heart rate (HR) data

HR was derived from electrocardiogram (ECG) which was simultaneously recorded with fMRI using an electrode included in the MRI compatible EEG system manufactured by Brain Products. The electrode was placed on the participant’s upper back as recommended by the manufacturer. Past work has shown that HR changes reflect autonomic system changes in response to aversive conditioning and can distinguish processes related to attentional orienting and active defense (Öhman, 2000; Öhman and Wiens, 2003). In addition, HR changes are sensitive to whether overt defensive (fear) responses are acquired, versus participants learning the contingencies without defensive mobilization (Hamm and Vaitl, 1996; Moratti and Keil, 2005).

### Data processing

#### fMRI data preprocessing

All fMRI analyses were performed in SPM (http://www.fil.ion.ucl.ac.uk/spm/). Preprocessing steps included slice timing, motion correction, and normalization to the Montreal Neurologic Institute (MNI) template. Normalized images were spatially-smoothed with a 7-mm full width at half maximum (FWHM) Gaussian kernel. This spatial smoothing step was omitted for the RSA analysis to better preserve spatial patterns. Global scaling was applied to remove the global signal from the BOLD time series (Desjardins et al., 2001). As a control, all analyses were repeated without global scaling, and the main results as reported below remained unchanged. The BOLD time series were then high-pass filtered with a cutoff frequency at 1/128 Hz. To address the potential problem of carry-over effects from US presentations into the subsequent trials, the main analyses below were also conducted using only trials with no immediately preceding US. Again, this did not affect the main findings reported in the results section, which reports analyses conducted on all trials except paired CS+ trials (i.e., trials in which the final 500 ms of the CS+ were accompanied by the loud scream).

#### Single-trial estimation of BOLD response

Single trial BOLD response was estimated on a trial-by-trial basis using the β-series method (Rissman et al., 2004, 2008). In this method, every stimulus was treated as a regressor in the general linear model (GLM). Rigid body movements were included as regressors of no interest. Solving the GLM yielded a β value for each trial in each voxel.

#### Trial-by-trial BOLD dynamics

To assess broad temporal dynamics across habituation and acquisition, we first divided each block into an early time period (*t* < 5.6 mins) and a late time period (*t* > 5.6 mins). This method of temporal demarcation using time-on-task, rather than trial counts, was to avoid bias toward a given stimulus type during acquisition. Single-trial β values for CS+ trials (those unpaired with the US) and CS– trials were separately averaged within each time period. A differential response was generated by subtracting the mean β values of CS– trials from the mean β values of the CS+ trials and subjected to statistical comparison using paired *t test*. Here, the differencing operation helped to minimize the impact of factors that might be time-dependent but were not related to the effects of fear learning. The phasic HR response, defined as initial HR deceleration, was similarly treated and included in the analysis as an autonomic index of defensive (fear) orienting to conditioned threat cues (Moratti and Keil, 2005).

If a region showed an early-versus-late period difference, it was defined as a region of interest (ROI), and its more refined temporal dynamics were further examined by smoothing the single-trial BOLD responses (β values) with a Gaussian smoother. The smoothed CS– response curve was subtracted from the smoothed CS+ response curve to yield the differential response curve for the same reason stated above. From this curve, temporal dynamic of BOLD activity in the ROI, especially significant changes in the CS+ versus CS– contrast, can be quantified.

#### Change-point analysis

Onset of trend change in the above differential response curve is a reflection of the CS+ related adaptation and thus has neurophysiological significance. Such change can be detected by combining cumulative sum (CUSUM) chart and permutation test (Taylor, 2000; Kass-Hout et al., 2012). To construct a CUSUM chart, each point in the CUSUM chart was calculated by cumulating the difference between current β contrast and the mean of all β contrasts onto the previous sum. Suppose that the values tend to be above the overall average during a period of time. The numbers added to the cumulative sum will be positive and the sum will steadily increase. In other words, an upward slope in the CUSUM chart indicates a period where the values tend to be above the overall average. Likewise, a downward slope in the CUSUM chart indicates a period of time where the values tend to be below the overall average. Any change in direction of the CUSUM chart indicates a shift in the average, thus a noticeable change in raw data. To determine if a change is statistically significant, a permutation procedure was performed. In this procedure, all the β contrasts were randomly reordered 1000 times, and the magnitude of change, which was calculated for each permutation as the difference between the maximum and the minimum of the cumulative sums, was extracted. The confidence level, calculated as the percentage of the magnitude of change in the permutation samples that was smaller than the original magnitude of change, was then compared to a threshold (95%) to determine if a change is significant. Once a significant change was detected, an estimation of when the change occurred, namely the change point, could be made. Here, the CUSUM estimator, a well-accepted estimator of change point, was taken as the estimation of change point, which corresponded to the maximum of cumulative sum chart.

For multiple ROIs, to test whether the change points were statistically different, we constructed the CUSUM chart for each subject and performed a one-way ANOVA on the estimated change points across ROIs. The question of whether the change points covaried across subjects was assessed by a principal component analysis (PCA). The lack of difference in the change points across multiple ROIs and a large variance explained by the first principal component were taken as evidence supporting the possibility that the set of ROIs may act in a concerted fashion to mediate conditioning dynamics at a network level.

#### Representational similarity analysis (RSA)

Temporal dynamics of CS+ and CS– evoked activities can be further studied using RSA, a MVPA method (Visser et al., 2011, 2013). To this end, we applied the β-series method to the BOLD time series before spatial smoothing, to maximally retain information at a finer spatial scale (Dunsmoor et al., 2014). For a given ROI, a vector was created from the β values to represent the spatial pattern in response to a stimulus; the length of the vector equals to the number of voxels in that ROI. To generate the time course of RSA, a sliding window approach was adopted, in which the time window was 50 trials in duration and the step size was two trials. Within each time window, the patterns for CS+ and for CS– were separately averaged, and then correlated with each other to assess pattern similarity. The correlation coefficients were Fisher-z transformed, averaged across subjects, and re-transformed back to correlation coefficients before being subtracted from 1 to yield the time course of dissimilarity between CS+ and CS– for a given ROI. Here, dissimilarity was used instead of similarity, in conformity with the standard practice in the field (Kriegeskorte et al., 2008). Temporally increased (decreased) dissimilarity is taken to suggest that the neural representations for CS+ and CS– become more (less) distinct over time.

To test whether pattern changes across ROIs were related, the slopes of the dissimilarity time courses were derived at the individual subject level for each ROI, and examined by a combination of correlation analysis and a PCA analysis. Functional significance of concerted changes in temporal dynamics of patterns was examined by correlating the score of the first principal component with the slope of the univariate amygdala adaptation time course.

#### HR analysis

The RR intervals was estimated from the ECG data and transformed into instantaneous HR (inverse of RR interval). The time range from 1-s prestimulus to 5-s poststimulus was divided into 1s bins, and each instantaneous HR was weighted proportionally to the fraction of the bin it occupied (Gatchel and Lang, 1973; Graham, 1980) to yield stimulus-locked HR change times series within a trial. Time courses of HR changes over habituation and acquisitions trials were similarly analyzed as above.

## Results

### HR analysis

**Figure 2.**
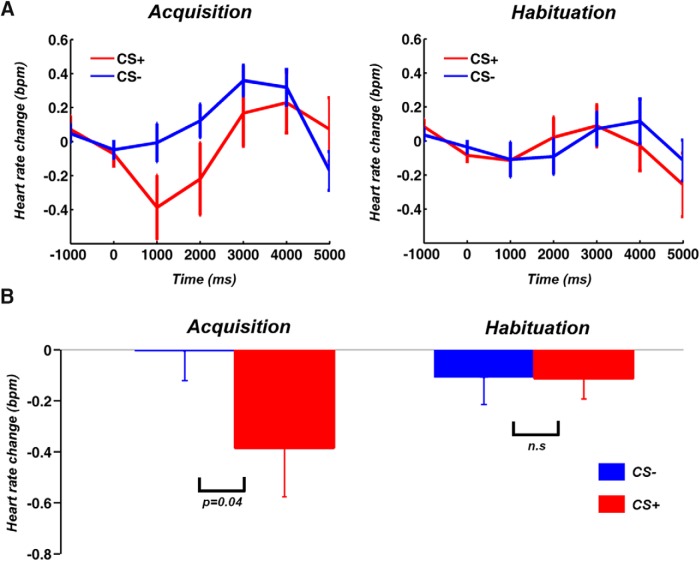
HR responses. ***A***, HR was more decelerated following CS+ compared to CS– during acquisition; no such difference was observed during habituation. ***B***, The HR difference during acquisition was statistically significant at *p* < 0.05. BPM, beat per minute.

Consistent with the extant literature (Moratti and Keil, 2005; Thayer et al., 2009), during acquisition, HR decelerated following CS+ (in this section, “CS+” refers to CS+ trials in which the CS+ was not paired with US), compared to CS– (Fig. 2*A*,*B*), demonstrating that participants acquired the contingencies of the experiment, and exhibited defensive orienting to the CS+; no such HR differences were observed during habituation.

### BOLD activation analysis

**Figure 3.**
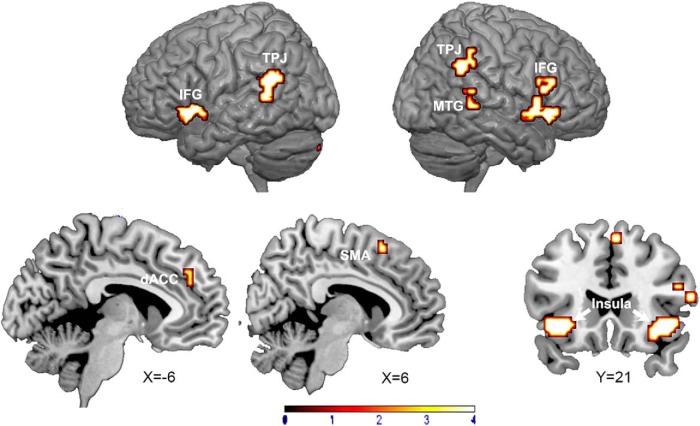
Statistical parametric maps showing regions that are more activated by CS+ than CS– during acquisition block (*p* < 0.05, FDR corrected, cluster size > 5).

As shown in Figure 3 and Table 1, brain regions showing higher activation for CS+ relative to CS– included bilateral insula, dACC, supplementary motor area (SMA), and the bilateral temporoparietal junction (TPJ); the amygdaloid bodies were not activated in this comparison.

**Table 1.** Regions activated in CS+ versus CS– contrast

Anatomical regions	MNI coordinates	*t* values/*z* values
STG (L)	-57 -45 21	7.04/4.76
Insula (R)	42 24 -6	6.09/4.38
IFG (L)	-36 24 -6	5.83/4.26
Insula(L)	-42 12 -3	4.97/3.85
TPJ (R)	60 -51 33	5.28/4.01
IFG (BA44, R)	60 18 15	5.03/3.88
MTG (R)	57 -39 0	5.02/3.88
IFG (BA48, R)	48 15 21	4.62/3.67
TPJ (R)	57 -42 45	4.52/3.62
IFG (BA45, R)	54 27 21	4.45/3.57
dACC	-3 39 36	4.60/3.66
SMA	6 21 54	4.60/3.66

STG: superior temporal gyrus; IFG: inferior frontal gyrus; TPJ: temporoparietal junction; MTG: Middle temporal gyrus; SMA: supplementary motor area.

### Early period versus late period analysis

**Figure 4.**
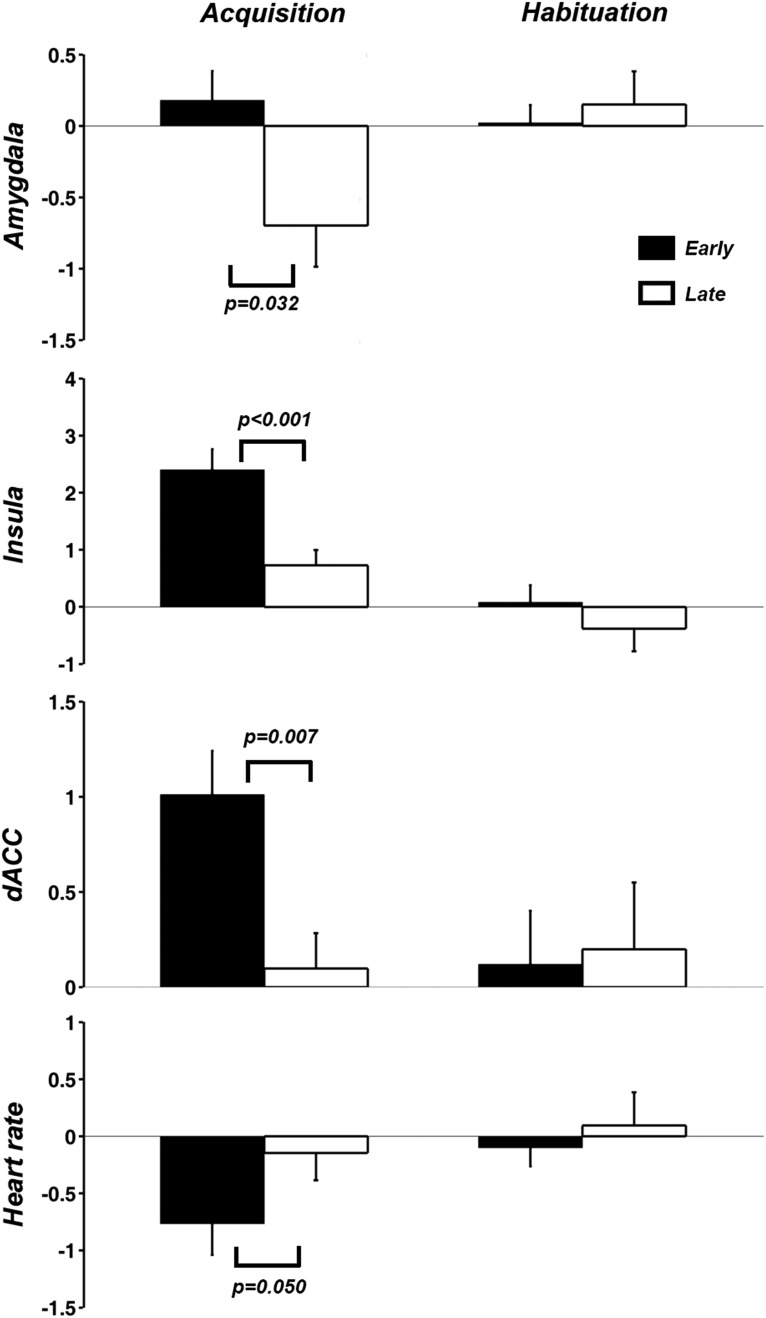
Early-versus-late period comparison. Left, Amygdala, insula, and dACC BOLD activity as well as HR deceleration exhibited significant late period CS+ relative to CS– decrease during the acquisition block. Right, No such differences were observed for the habituation block (*p* = 0.72 for amygdala, 0.26 for insula, 0.78 for dACC, and 0.42 for HR).

The acquisition block was divided into two halves with the first half and the second half referred to as early time period and late time period respectively. The 12 regions in Table 1, along with the right amygdala (center coordinate: 21, -3, -16) that appeared in the US activation map at *p* < 0.05 FDR, were analyzed for early-versus-late differences. Single-trial brain responses to CS+ and CS– were estimated using the β-series method. For each of the 13 regions, differential activities (CS+ minus CS–) in the early and late period of acquisition were compared, and significant differences were found in right amygdala, right insula, and left dACC (Fig. 4). The differences in dACC and insula were significant at *p* < 0.05 after applying FDR multiple comparison correction. HR differences between CS+ and CS– were also significantly diminished in the late period. During habituation, no differences between the early time period and late time period were found for any of the dependent variables.

### Time-course analysis

**Figure 5.**
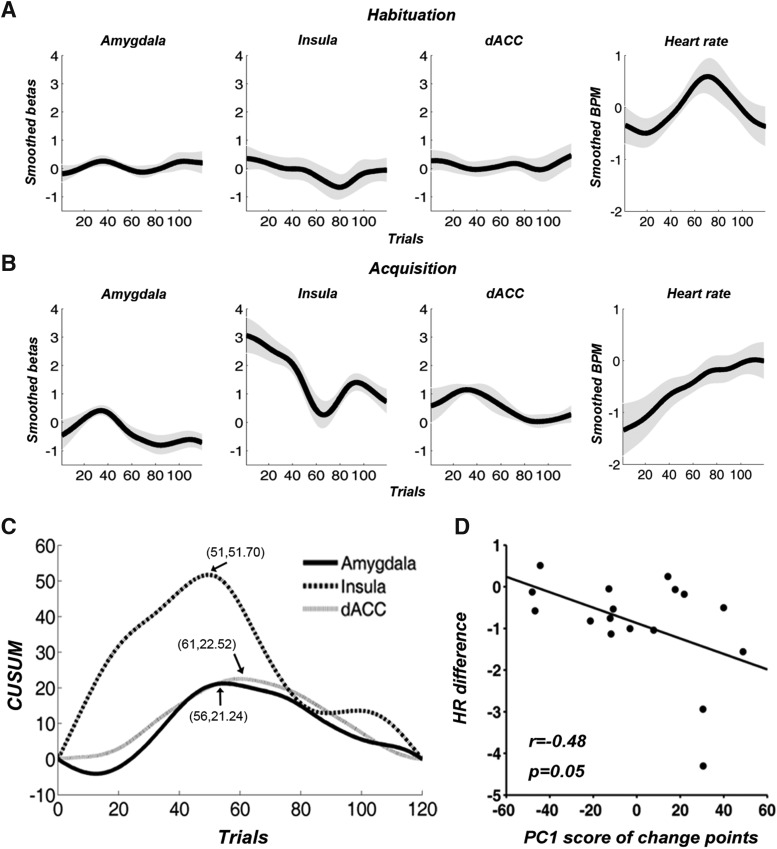
Temporal dynamics of brain activity in three ROIs and HR. ***A***, During habituation, time courses of differential BOLD activity were flat in the three ROIs, and there was no systematic trend in differential HR. ***B***, During acquisition, a general declining trend for BOLD as observed for each ROI, and HR difference also diminished. ***C***, Three change points detected by CUSUM chart were marked by the arrows and the numbers in the bracket gave the corresponding trial number and the cumulative sum. ***D***, HR difference (CS+ minus CS– over acquisition) was negatively associated with the score of the first principal component (PC1) from the three change points of the three ROIs. Gray shade in ***A***, ***B*** represents standard error of the mean.

Defining the three regions in Figure 4 as ROIs, their single-trial β values were smoothed over time. A differential time course was then created by subtracting CS– response time course from CS+ response time course. As shown in Figure 5*A*,*B*, for the habituation block, the time courses were essentially flat, hovering around zero, whereas for the acquisition block, a generally declining tendency was observed for the ROIs. For differential HR, there was no systematic trend during habituation, whereas during acquisition, HR difference between CS+ and CS– systematically declined (Fig. 5*A*,*B*).

The neural time courses of the three ROIs in Figure 5*B* were further analyzed using the change-point analysis to quantitatively detect the change point at which the decline of differential BOLD response occurred (Weinberger et al., 2006). As shown in Figure 5*C*, the estimated change points were the 56^th^ trial, the 51st trial, and the 61st trial for the amygdala, insula, and dACC, respectively. To test whether these change points were statistically robust, a permutation procedure was conducted, and all three change points were statistically significant with larger than 95% confidence. ANOVA (18 subjects × three ROIs) applied to the change points from the three ROIs obtained at the individual subject level indicated that there were no significant differences in the three change points from the three ROIs (*F*_(2,51)_ = 2.84, *p* = 0.10). PCA further demonstrated that the first principal component explained 50% of the variance, suggesting that the three change points covaried across subjects (if the three variables were independent then the variance explained by the first PCA component would be 33%). Functional significance of this concerted variation in change points was examined by their association with physiologic changes (HR difference between CS+ and CS– over the acquisition session). As shown in Figure 5*D*, the score of the first principal component was significantly negatively correlated with HR difference, indicating that individuals with stronger HR orienting (greater HR deceleration) displayed later BOLD adaptation in the salience network.

### Time course of neural representation change

**Figure 6.**
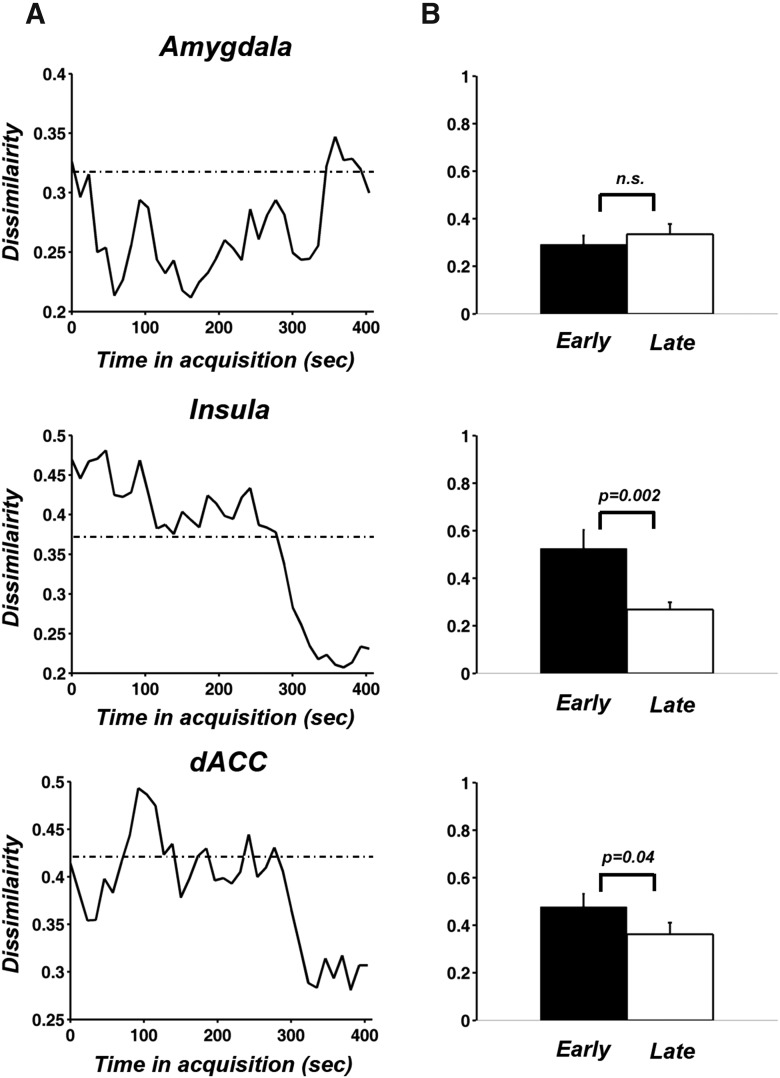
Time course analysis of multivoxel patterns evoked by CS+ and CS–. ***A***, The time courses of RSA dissimilarity between CS+ and CS– for the three ROIs. The dashed line in each plot indicated the averaged RSA dissimilarity value of that ROI in habituation. ***B***, A paired *t* test was performed to compare mean dissimilarity of the first 10 points and the last 10 points of the RSA dissimilarity curves in ***A***.

The univariate BOLD analysis applied above defines one facet of the adaptive neural dynamics. To take advantage of the information contained in activity patterns across voxels in a ROI, and to investigate how fear conditioning alters the neural representation of the CS, we applied RSA to examine the temporal change of multivoxel patterns evoked by CS+ and CS– (Visser et al., 2011, 2013; Fig. 6). The decreasing dissimilarity between the patterns evoked by CS+ and CS– in insula and dACC further strengthens the evidence for adaptation; that is, the response patterns of these higher order brain regions to CS+ and CS– became less distinctive toward the end of the acquisition. In the amygdala, voxel patterns dissimilarity between CS+ and CS– trials did not exhibit clear change during acquisition, unlike univariate BOLD magnitude.

**Figure 7.**
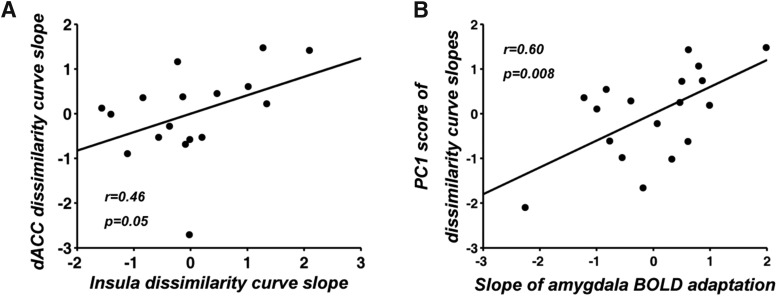
Analysis of pattern adaptation slopes. ***A***, Slopes of RSA dissimilarity curves from insula and dACC were positively correlated. ***B***, The first principal component explained 73% of the variance of the dissimilarity slope data and its score was positively correlated with the rate of amygdala adaptation.

To what extent changes of voxel patterns in insula and dACC were related was examined by comparing the slopes of the two dissimilarity time courses derived at the individual subject level. Figure 7*A* showed that the two slopes were significantly correlated (*r* = 0.46, *p* = 0.05), indicating that the rate of pattern adaptation in insula and dACC behaved in a concerted fashion. This was further supported by a PCA analysis where the first principal component explained 73% of the total variance (if the two variables were independent then the variance explained by the first PCA component would be 50%). Furthermore, as shown in Figure 7*B*, the first principal component score exhibited a positive correlation (*r* = 0.6, *p* = 0.008) with the slope derived from the univariate amygdala adaptation time course, suggesting that faster amygdala adaptation predicted faster decrease in representational distinctness between CS+ and CS– multivoxel patterns in insula and dACC.

## Discussion

We investigated the temporal dynamics of neural activity during classical fear conditioning using fMRI. Relative to previous studies, our experimental design incorporated two key considerations: (1) a substantial number of trials were included to allow the testing of the full development of conditioning dynamics and (2) a simple visual feature (grating orientation) was used to discriminate the otherwise identical CSs to allow identification of neural representational changes that were specifically related to the changing distinctiveness between two CSs due to aversive learning. Applying single-trial BOLD analysis and multivoxel pattern analysis, we found that (1) the BOLD contrast of CS+ versus CS– across the entire acquisition block exhibited heightened BOLD for the CS+ in dACC and insula but not in the amygdala; (2) relative to the CS–, single-trial BOLD responses to the CS+ in the amygdala substantially decreased over the course of the acquisition block; (3) dACC and insula also showed declined differential response over the course of acquisition; (4) amygdala, insula and dACC exhibited concerted adaptation dynamics in terms of timing in activity decline, suggesting coordination at a network level; (5) the voxel patterns evoked by CS+ and CS– in dACC and insula became increasingly less distinguishable over the course of acquisition with correlated rates of decrease; and (6) BOLD magnitude adaptation dynamics in the amygdala predicted autonomic (HR) changes as well as representational dissimilarity dynamics in insula and dACC.

These findings support the hypothesis that the amygdala, important in the initial acquisition of CS-US contingencies (the first four CS+ trials were paired with US), is not involved in the sustained discrimination of CS+ and CS– trials. They further demonstrated that insula and dACC also became progressively less engaged in such discrimination processes. These results, in addition to yielding further insight into amygdala adaptation, support the notion that the salience network, which includes the amygdala, insula and dACC, plays a significant role initially in discriminating threat and safety cues, but with a substantial amount of trials, a long initial habituation block, and a simple feature serving as the CS+, this role diminished over time. The observation that BOLD magnitude reduction was accompanied by reduced pattern dissimilarity appears to be at odds with studies that did not use a habituation block at the beginning of the experiment, where magnitude reduction was reported to concur with heightened pattern dissimilarity, interpreted as reflecting the formation of more refined and sparser representations (Bach et al., 2011). Our findings are, however, consistent with the possibility that sustained exposure to contingencies prompts broader changes in brain network configuration, including increased involvement of posterior sensory cortex (Miskovic and Keil, 2013).

Studies in rodents have provided ample evidence suggesting that the amygdala is a critical component of the neural circuitry underlying fear conditioning. Plastic changes in the lateral amygdala have been proposed as one mechanism contributing to forming CS-US associations in the brain (Li and McNally, 2014). Acquiring defensive responses associated with CS-US pairing may increasingly involve changes in structures such as the thalamus, sensory cortices, basal ganglia, motor system structures, prelimbic (PL) cortex, hippocampus and periaqueductal gray, mediated by the medial central amygdala through its dense connections with these brain networks (Ciocchi et al., 2010; Johansen et al., 2010). Once a CS-US association has been acquired, a decline in amygdala activation may occur at the macroscopic level (Büchel and Dolan, 2000), unless subsequent changes in CS-US contingency take place. This led to the hypothesis that the amygdala mediates the initial acquisition of the CS-US association, but not its maintenance (Pape and Pare, 2010). It is worth noting that amygdala adaptation is not seen in studies that adopt complex aversive cues such as naturalistic scenes displaying burn victims, car accidents, and attack scenes (Liu et al., 2012; Bradley and Lang, 2015). These considerations support the notion that amygdala adaptation is a signature of repetitive exposure to relatively simple stimuli such as electric shock, loud noise, and experimental pain.

Our single-trial BOLD activation analysis in the amygdala agrees with the notion discussed above. Given the lack of amygdala activation in the CS+ versus CS– contrast, we defined the (right) amygdala ROI based on US-related activation. The temporal response profile of the left amygdala, which showed no US or CS related differences at the block level, was also explored and no significant temporal change was found (results not shown). This hemispheric lateralization pattern is consistent with previous literature showing stronger adaptation in the right rather than the left amygdala (Phillips et al., 2001; Wright et al., 2001; Wedig et al., 2005; Denny et al., 2014). In a similar vein, right amygdala lesions specifically impair the expression of defensive responses evoked by visual scenes (Angrilli et al., 1996), whereas the left amygdala has often been related to conditioned responses evoked by verbal information (Funayama et al., 2001). The present single-trial BOLD activation analysis also demonstrated relative amygdala deactivation for the CS+, compared to the CS–, during the late portion of the acquisition block. This finding replicates similar observations in previous neuroimaging studies of fear conditioning (Büchel et al., 1998; Straube et al., 2007). Several conceptual and neurophysiological notions are consistent with this finding. First, studies where computational models of fear conditioning were fit to fMRI data have shown that amygdala BOLD tracks associability (the previous information content of a conditioned cue), rather than valence or saliency of the CS+ (Li et al., 2011). In our paradigm, the initial four CS+ trials during acquisition were paired with the US, and thus had 100% contingency to establish a strong associative relation between the CS+ and the US. It would not be unexpected then that the remaining CS+ data included in the analysis reflected a situation in which associability (surprise value) of the CS+ is already greatly diminished, prompting diminished amygdala engagement. Second, it is well established that initial presentation of the CSs in a safe context (the habituation block) reduces subsequent associative learning, i.e., latent inhibition (Lubow, 1973). Because of our interest in the temporal evolution and adaptation, we used a long initial habituation block, which may have resulted in delayed and attenuated acquisition of the CS+, due to pre-exposure before the beginning of acquisition. If amygdala BOLD response to the CS+ reflects a transient associative process, then an experimental design such as ours should result in relatively diminished CS+ evoked BOLD responses, as observed. Finally, amygdala deactivation, along with the reduced activation in dACC and insula, may represent a part of the brain’s adaptation to predictable aversive stimulation. In a study of experimental pain, Petrovic et al. (2004), manipulating the context preceding a noxious event, observed anticipation related amygdala deactivation and hypothesized that it was part of a coping strategy to reduce nociceptive sensitivity to the impending painful stimulation. Future work using experimental manipulations of these factors, in conjunction with single-trial BOLD activation dynamics, is a key to adjudicate between these notions.

A growing body of work has established that BOLD fluctuations in the amygdala, dACC, and insula covary, forming the so-called salience network, involved in the detection and integration of salient events or stimuli (Seeley et al., 2007). Although insula and dACC have been discussed in the context of fear conditioning, they are often treated as separate entities, rather than as key nodes of a brain network. For example, the discussion on the role of dACC in fear conditioning is often conducted in the context of its rodent homolog, the PL region (Milad and Quirk, 2012), which is known to play an important role in the acquisition of conditioned defensive responses (Powell and Ginsberg, 2005; Vidal-Gonzalez et al., 2006; Adhikari et al., 2015). Similarly, the insula’s role is often emphasized by its consistent activation by conditioned threat (Sehlmeyer et al., 2009), and by its covariation with self-reported emotional arousal (Phelps et al., 2001; Critchley et al., 2002; Sarinopoulos et al., 2010). The present finding that the trial-by-trial BOLD dynamics in the amygdala, dACC, and insula share significant temporal characteristics (e.g., the three change points in the BOLD time courses from the three ROIs covaried across subjects), supports the notion that aversive conditioning engages these structures as a network, paralleling tasks that tap into salience processing.

In addition to univariate activation analysis, multivoxel patterns offered another opportunity to examine the temporal dynamics of the brain’s response to conditioned threat at the level of neural representations. Previous studies applying RSA to fear conditioning data have shown that associative learning is accompanied by increased similarity of BOLD-MRI patterns over consecutive trials within the same condition (Visser et al., 2011). Fear learning also modulates category-level representations of object concepts (Dunsmoor et al., 2014), and changes the neural response patterns that correlate with the enhancement of fear memory consolidation (Visser et al., 2015). In the present study, the representational dissimilarity between brain responses to CS+ and CS– decreased toward the later part of acquisition in dACC and insula, paralleling the declining activation in these structures obtained from univariate analysis. Importantly, the fact that the rate of RSA dissimilarity decreases in dACC predicted that in insula and vice versa, suggests that the adaptation dynamics at the neural representation level may be coordinated across multiple nodes of the salience network. These pattern analysis results cannot be explained simply in terms of reduced activation. In fact, an activation bias account of our findings, as demonstrated by Larocque et al. (2013), would predict the opposite effect, namely, less activation leads to larger dissimilarity because two random patterns have low correlation (high dissimilarity).

The lack of pattern similarity change in the amygdala may reflect that only a relatively low number of voxels is available for analysis and a reliable MVPA analysis requires a reasonable number of voxels. Because different subregions of the amygdala may have different representational dissimilarity dynamics, simply increasing the number of amygdala voxels used in the RSA analysis did not affect the outcome of the analysis. The low signal-to-noise ratio (SNR) in amygdala is another factor (Boubela et al., 2015). Applying univariate noise normalization (Visser et al., 2015), however, does not improve the SNR. In one previous study, Bach et al. (2011) were able to differentiate multivoxel amygdala activities between CS+ and CS– in a small sample of six participants. The longer ITI used in that study may have facilitated the development of discriminative patterns in amygdala (Visser et al., 2016). Future studies with finer spatial resolution are needed to further elucidate the nature of neural representational changes in amygdala during fear acquisition.

The temporal profile of amygdala adaptation, rather than amygdala mean activation, has been shown to possess substantial predictive value. For example, amygdala adaptation is negatively correlated with trait anxiety (Hare et al., 2008), severity of autism (Kleinhans et al., 2009), and genetic factors linked to depression and anxiety (Fisher et al., 2009; Lonsdorf et al., 2011). Furthermore, it has been reported that indices of amygdala adaptation showed higher within-subject reliability than standard metrics of the mean response amplitude, in an emotional face perception task (Plichta et al., 2014). Functionally, adaptation of amygdala responses has been linked to better management of limited resources by diminishing orienting/attention responses to stimuli that are no longer salient to the organism (Phillips et al., 2001; Wright et al., 2001). Our observation that the rate of adaptation of univariate BOLD responses in amygdala predicted neural representational changes in dACC and insula enriches this line of research and provides additional evidence that aversive learning may be mediated by the large-scale salience network rather than by a single brain area such as the amygdala. Similar ideas have appeared in a previous study showing that insula, cingulate cortex, and amygdala exhibited concerted activity in response to stimuli associability (Li et al., 2011).

The diminished role of the salience network with time in the acquisition block may be accompanied by plastic changes in other brain networks mediating the processing of CS and US. For example, brain regions outside the salience network become increasingly involved in selective sensory analysis of and motor response preparation to the CS+. Such increasing posterior involvement has been well documented for visual cortical structures (Miskovic and Keil, 2012; Petro et al., 2017). Future work may relate adaptation in the salience network to suitable metrics of visuo-cortical changes to address the relationship between these contrasting phenomena.

## Synthesis

Reviewing Editor: Daniela Schiller, Mount Sinai School of Medicine Decisions are customarily a result of the Reviewing Editor and the peer reviewers coming together and discussing their recommendations until a consensus is reached. When revisions are invited, a fact-based synthesis statement explaining their decision and outlining what is needed to prepare a revision will be listed below. The following reviewer(s) agreed to reveal their identity: Vishnu Murty, Erno Hermans.

The authors study is nicely designed and has an elegant analysis plan that shows different time courses of conditioned responses across regions, and shared information across these timecourses. This is a novel approach that is comprehensive and advances the field.

In particular, this paper addresses the interesting question of why amygdala responses attenuate so rapidly during fear conditioning experiments. The findings are comparable to previous work, but offer more insight into the temporal dynamics of habituation of amygdala responses during fear conditioning.

The authors have adequately addressed the concerns raised in the first round of reviews. This revised manuscript also better integrates previous findings on this topic.

There is one issue that remains. Even though the temporal dynamics of habituation/adaptation of the differential amygdala response seems similar to that of other salience network regions, there is a notable difference: as pointed out in the previous review, the differential amygdala response is close to zero early in acquisition, and appears to dip consistently below zero in later stages. In contrast, in other salience network regions, an initial positive differential response gradually returns to zero. The authors convincingly show that the negative differential response in the amygdala in later stages is not an artifact of global scaling. However, they do not discuss it further. If the authors agree that this negative differential response in the amygdala is a true finding - what implications does this have for their interpretation of the data, and for the alleged similarity of the temporal dynamics of amygdala with other salience network regions?
